# The Dynamic Trends of HIV Prevalence, Risks, and Prevention among Men Who Have Sex with Men in Chongqing, China

**DOI:** 10.1155/2014/602719

**Published:** 2014-03-25

**Authors:** Gang Zeng, Liangui Feng, Lin Ouyang, Rongrong Lu, Peng Xu, Guohui Wu, Fan Lu

**Affiliations:** ^1^National Center for AIDS/STD Control and Prevention, Chinese Center for Disease Control and Prevention, Beijing 100026, China; ^2^Chongqing Center for Disease Control and Prevention, Chongqing 400042, China

## Abstract

*Objective*. This study was to characterize the continuously changing trends of HIV prevalence, risks, sexual behaviors, and testing behaviors among men who have sex with men (MSM) in Chongqing, China. *Methods*. Five consecutive cross-sectional surveys were conducted among MSM in 2006, 2008, 2010, 2012, and 2013. Testing for HIV and syphilis was performed, and HIV risks, sexual behavior, prevention, and HIV testing behavior were collected using the same questionnaire. *Results*. HIV prevalence increased from 13.0% to 19.7% from 2006 to 2013 (*P* = 0.004), with an increase of 1.0% per year. Syphilis prevalence peaked in 2008 with a positive rate of 11.6% and then experienced a sharp drop to 2.8% in 2012 and 2.9% in 2013. Percentage of those who ever received HIV testing in the last year increased from 17.0% to 43.3% (*P* < 0.001); condom use at the last anal intercourse and reported consistent condom use in the last 6 months increased from 51.8% to 71.0% (*P* < 0.001) and from 24.7% to 47.9% (*P* < 0.001), respectively. *Conclusions*. HIV continued to spread among MSM in Chongqing even when a decline in prevalence of syphilis and increase in awareness rate, condom use, and HIV testing seeking behaviors seemed to occur.

## 1. Introduction

Unprotected sex among men who have sex with men (MSM) is the main route of HIV transmission in many countries and regions that were first affected by the HIV/AIDS epidemic, such as North America, Western Europe, and Australia [1]. Over the past years, HIV infection rate among MSM is on the decline in some cities in the United States [2]. On the contrary, HIV infection rate is increasing obviously among MSM in China [3, 4]. According to the national estimations of the HIV/AIDS epidemic in China, the proportion of HIV/AIDS cases infected via homosexual contact increased from 7.3% in 2005 to 13.0% in 2011 [5, 6]. It was estimated that 29.4% of new HIV infections in 2011 were transmitted via homosexual contact [6]. In 2008, a survey was conducted among 18,101 MSM in 61 cities in China and indicated an overall HIV prevalence of 5.1% among MSM. In some cities, HIV infection rate exceeded 10% among MSM [7, 8]. Chongqing is a city affected most heavily by homosexual transmission. The first HIV infection via homosexual contact was reported in Chongqing in 2005. Since then, the proportion of new HIV/AIDS cases via homosexual contact has shown a rapid upward trend [9], reaching 22.2% from January to June 2013, which is 0.8% higher for the same period in 2012. Understanding the trends of HIV/AIDS epidemics is crucial to identify priorities, revise strategies, and allocate resources in the response to HIV. In Chongqing, serological and behavioral surveillance has been conducted among MSM since 2006, and sound historical data have been accumulated [10, 11]. This study aims to capture the changes in the trends of the HIV/AIDS epidemic and risk behaviors of the MSM population based on the surveillance data from 2006 to 2013 in Chongqing and provide scientific evidence for the development of strategies and measures in the city's response to HIV.

## 2. Methods

### 2.1. Study Population

Men who were at least 18 years of age and had sexual intercourse with at least 1 male during the previous year were eligible to participate in the study, regardless of their residence, history of HIV testing, serostatus, or treatment status.

### 2.2. Recruitment

Under the guidance of the National Protocol for HIV Sentinel Surveillance, a national MSM sentinel site was established in Chongqing and began operations in 2006. The period of surveillance was from April to June every year. A number of recruitment strategies were adopted, including receiving referrals from nongovernment organizations, adopting the snowball method, and using online recruitment. The numbers of participants were 561, 602, 400, 390, and 376 in 2006, 2008, 2010, 2012, and 2013, respectively.

### 2.3. Measures

The survey site was located in a STI/HIV clinic of Chongqing CDC. After obtaining informed consent from study subjects, trained investigators administered a questionnaire to collect information on the study subjects' demographic characteristics, homosexual behaviors, heterosexual behaviors, commercial sex, and access to HIV testing and intervention. Blood samples were taken from subjects for HIV and syphilis testing. HIV infection was screened with enzyme-linked immunosorbent assay (ELISA; Zhuhai Livzon Diagnostics Inc., China). Subjects with a positive result in the first test were retested with another ELISA reagent (Beijing Wantai Biological Pharmacy Enterprise Co., Ltd., China). HIV infection was diagnosed if both tests were positive. For syphilis testing, ELISA assay (Zhuhai Livzon Diagnostics Inc., China) and TRUST assay (Shanghai Rongsheng Biological Pharmacy Enterprise Co., Ltd., China) were used, and there would be a confirmed diagnosis if both ELISA and TRUST positive could be diagnosed syphilis positive. However, only ELISA assay was used in 2006 and 2008.

### 2.4. Statistical Analysis

Data were entered into a web-based electronic database designed by the National HIV Sentinel Surveillance Working Group. Upon completion of data inputs, data were then exported to the SAS software for statistical analysis (SAS Institute Inc., USA, version 9.3). Demographic characteristics were presented and compared using chi-square test for different rounds of samples. Observed trends in HIV and syphilis prevalence, as well as a number of sexual risk behaviors, were analyzed with chi-square trend test (Cochran-Armitage method). Since the study samples across the study period were different in age proportion, trends were also examined separately for participants aged between 18 and 24 years, 25 and 34 years, and those aged over 45 years. All reported *P* values were examined at a 2-tailed test. *P* < 0.05 was considered as a statistically significant difference.

## 3. Results

### 3.1. Study Participants

A total of 2329 MSM were enrolled in the sentinel surveillance surveys: 561 participated in 2006, 602 in 2008, 400 in 2010, 390 in 2012, and 378 in 2013 (Table 1). The mean age (SD) was 31.8 (9.9), 26.0 (6.8), 26.7 (6.9), 27.4 (6.4), and 27.4 (7.0) in 2006, 2008, 2010, 2012, and 2013, respectively. Subjects in 2006 were older than those in other years. Most subjects were single, accounting for 83.1%. 23.7% of subjects were migrants. In 2008, 38.0% of subjects (the highest figure across the years covered by the study) were migrants. 62.3% of subjects have received education for at least 13 years. The education level was obviously higher among subjects surveyed in 2010 than those surveyed before 2010. There are significant differences in demographic characteristics of subjects across the survey years (*P* < 0.001).

### 3.2. Dynamic Trends of HIV and Syphilis

HIV prevalence among the study participants showed an upward trend and increased from 13.0% to 19.7% from 2006 to 2013 (*P* = 0.004), with an increase of 1.0% per year. After age stratification, HIV prevalence significantly increased from 6.3% to 19.6% among MSM aged 18–24 (*P* < 0.001) and had a modest increase from 13.7% to 19.4% among MSM aged 25–34 (*P* = 0.085). No significant trend was observed among those over 35 years old (*P* = 0.18). However, syphilis prevalence peaked in 2008 with a positive rate of 11.6% and then sharply dropped to 2.9% in 2013. The decreasing trend could be observed in all three age groups and subgroups by HIV infection status. In addition, both HIV infection rate and syphilis infection rate increased by age, with MSM over 35 having the highest HIV/syphilis prevalence in all survey years. HIV positive cases had higher syphilis prevalence compared to HIV negative cases over the study period, except in the year 2012. See Table 2.

### 3.3. Sexual Behavior and Trends of Reported Condom Use

Percentage of receiving HIV testing in the last year increased from 16.8% to 43.1% (*P* < 0.001). Reported condom use at the last anal intercourse and reported consistent condom use in the last 6 months increased from 51.9% to 71.0% (*P* < 0.001) and from 24.7% to 47.9% (*P* < 0.001), respectively. HIV/AIDS awareness rate rose from 87.0% to 98.0% (*P* < 0.001). The proportion of subjects ever having had sex with women decreased from 33.0% to 8.8%. Just like the rate of condom use with men, the rate of condom use with women was also on the increase. The rate of condom use at last sex increased from 30.8% to 51.5%. The rate of consistent condom use in the past six months increased from 21.6% to 45.5%. See Table 3.

## 4. Discussion

Despite huge amounts of effort in HIV prevention and control, this analysis has shown that there has been a significant increase in HIV prevalence among MSM population in Chongqing from 2006 to 2013, coinciding with the general trend in China [4, 12]. This rise in prevalence is mainly due to the rapid increase in HIV infection among MSM individuals aged 18–24 years, which resulted in the fact that all age groups had similar HIV prevalence in 2013 although there was a huge gap (6.3% for 18–24, 13.7% for 25–34, and 17.7% for over 35) in 2006. Previous study indicated that younger MSM are more likely inclined to have multiple sex partners and engage in more complicated sexual networks and older MSM may prefer to have young MSM partners [13]. The sexual intercourse between older MSM with higher HIV prevalence and young MSM could be a contributing factor to the increase of HIV prevalence among young MSM. HIV prevalence among older MSM has stabilized in recent years, as a result of more people having access to ART treatment and decreasing number of MSM dying from AIDS. However, it is also likely that the HIV prevalence may have been overestimated. Firstly, in all rounds of surveys, HIV positive cases were defined as two ELISA positives, which may include some false positives. Secondly, the participants were conveniently sampled. One meta-analysis indicated that the unprotected anal intercourse prevalence was significantly lower in studies that used random or systematic sampling methods compared with studies that used convenience sampling, as more high-risk MSM could have been recruited, resulting in higher estimate of HIV prevalence [14].

Our results suggest that there is a significant increase in condom usage and more MSM are willing to seek HIV testing. This could be partly a result of the numerous MSM intervention projects implemented by the local government, as well as the work of all the MSM nongovernment organizations in Chongqing these years [10]. The positive trend could also be seen in other studies in China [15, 16]. However, the current level of condom usage is still very low and could be a reason for the elevated prevalence. Many countries across the world are struggling to reduce unprotected sexual behavior among MSM [17, 18] and to reduce HIV infection among MSM, additional effort is needed to expand HIV prevention programs, taking into consideration of the biomedical, behavioral, and structural aspects of the epidemic, which could lead to possible improvements in safe sexual behavior, HIV testing seeking and access to care, and subsequently a decline of HIV prevalence in the long run.

There are a few reasons for the discrepancy between HIV prevalence increase and condom use rate. Firstly, prevalence reflects joint action of incidence and mortality. Prevalence might go up when both incidence and mortality go down. In fact, with expansion of ART, the mortality does decrease significantly in recent years in China [19]. In general, population incidence can reflect epidemic trends most directly. The surveillance of incidence, however, has not been implemented widely due to constraints of laboratory capacity, staffing, timing, and resources. Many studies adopted prevalence from continuous cross-sectional surveys to estimate the trends of HIV/AIDS epidemic [20, 21]. Secondly, Sentinel surveillance is grounded on anonymous unlinked survey. The duplication of the recruitment over the years might have overestimated condom use rate and HIV testing rate.

The analysis indicated a significant decline in syphilis infection rate in Chongqing, which could also be seen in other Chinese cities [22]. Since TRUST reagents were not used for syphilis screening in 2006 and 2008, the use of single ELISA might produce a high proportion of false positive cases. Excluding data from these two years, an obvious decline was still observed for syphilis infection rate after 2010. Additionally, the proportion of subjects self-reporting sexually transmitted diseases in the previous year was also on the decline, consistent with syphilis testing results and coinciding with the self-reported increasing rate of condom use. This indicated that safer sex could be effective in preventing STDs but needed to be confirmed by more data. Results showed that the rate of syphilis infection and self-reported STD was higher among HIV-positive subjects than among HIV-negative subjects, consistent with other studies [23]. The high rate of coinfection justified the necessity of screening in both HIV positive cases and STD patients.

There are several limitations in this analysis. Firstly, the sentinel surveillance relied on convenience-based samples and the participants were recruited through several different methods in every round. Since the characteristics of samples in each survey showed statistically different results, stratified analysis was performed to minimize the effect of the change in population structure.

Secondly, changes in HIV tests may also influence trends. HIV antibody tests have improved over the years. New generation assays are more accurate and the rate of false-positive results that may skew prevalence becomes lower. Thirdly, Reporting bias due to desirability is more likely to affect the assessment of trend data. Since incidence is the best indicator of HIV trend in a short period, trend analysis based on incidence estimates was strongly recommended for future study.

## Figures and Tables

**Table 1:** Demographics of Participants among Men Who Have Sex with Men in Chongqing.

Demographics	2006		2008		2010		2012		2013		Total	
	*N*	*n* (%)	*N*	*n* (%)	*N*	*n* (%)	*N*	*n* (%)	*N*	*n* (%)	*N*	*n* (%)
Age (years)												
18–24	561	158 (28.2)	602	322 (53.5)	400	184 (46.0)	390	144 (36.9)	376	153 (40.7)	2329	961 (41.3)
25–34	561	211 (37.6)	602	205 (34.1)	400	172 (43.0)	390	201 (51.5)	376	175 (46.5)	2329	964 (41.4)
≥35	561	192 (34.2)	602	75 (12.5)	400	44 (11.0)	390	45 (11.5)	376	48 (12.8)	2329	404 (17.3)
Marital Status												
Single	561	416 (74.2)	602	516 (85.7)	391	341 (87.2)	390	335 (85.9)	376	320 (85.1)	2320	1928 (83.1)
Married or divorced	561	145 (25.8)	602	86 (14.3)	391	50 (12.8)	390	55 (14.1)	376	46 (14.9)	2320	382 (16.5)
Residence												
Local	561	441 (78.6)	602	373 (62.0)	398	316 (79.4)	390	326 (83.6)	376	319 (84.8)	2327	1775 (76.3)
Non-Local	561	120 (21.4)	602	229 (38.0)	398	82 (20.6)	390	64 (16.5)	376	57 (15.2)	2327	552 (23.7)
Education (years)												
0–9	561	137 (24.5)	602	97 (16.2)	399	14 (3.6)	390	18 (4.6)	376	28 (7.4)	2328	294 (12.6)
10–12	561	198 (35.3)	602	174 (28.8)	399	91 (22.8)	390	49 (12.6)	376	71 (18.9)	2328	583 (25.0)
≥13	561	226 (40.2)	602	331 (55.0)	399	294 (73.7)	390	323 (82.8)	376	277 (73.7)	2328	1451 (62.3)

**Table 2:** Dynamic trends of HIV, Syphilis, and other sexually transmitted diseases among men who have sex with men in Chongqing.

Infections/Diseases	2006		2008		2010		2012		2013		*P* value*
	*N*	*n* (%)	*N*	*n* (%)	*N*	*n* (%)	*N*	*n* (%)	*N*	*n* (%)	
HIV	561	73 (13.0)	602	98 (16.3)	400	50 (12.5)	390	76 (19.5)	376	74 (19.7)	0.004
Age (years)											
<25	158	10 (6.3)	322	39 (12.1)	184	22 (12.0)	144	26 (18.1)	153	30 (19.6)	<0.001
25–34	211	29 (13.7)	205	29 (14.1)	172	19 (11.0)	201	36 (17.9)	175	34 (19.4)	0.085
≥35	192	34 (17.7)	75	20 (26.7)	44	9 (20.5)	45	14 (13.1)	48	10 (20.8)	0.18
Syphilis	561	54 (9.6)	602	70 (11.6)	400	25 (6.3)	390	11 (2.8)	376	11 (2.9)	<0.001
Age (years)											
<25	158	6 (3.8)	322	29 (9.0)	184	12 (6.5)	144	3 (2.1)	153	4 (2.6)	0.044
25–34	211	19 (9.0)	205	26 (12.7)	172	9 (5.2)	201	5 (2.5)	175	3 (1.7)	<0.001
≥35	192	29 (15.1)	75	15 (20.0)	44	4 (9.1)	45	3 (6.7)	48	4 (8.3)	0.056
HIV and syphilis coinfection	73	15 (20.5)	98	29 (29.6)	50	7 (6.5)	76	2 (2.1)	74	9 (12.2)	<0.001
Self-report STD diagnosis in the last year	561	112 (20.0)	602	122 (20.3)	400	22 (5.5)	390	11 (2.8)	376	9 (2.4)	<0.001
Age (years)											
<25	158	34 (21.5)	322	58 (18.0)	184	11 (6.0)	144	6 (4.2)	153	3 (2.0)	<0.001
25–34	211	53 (25.1)	205	46 (22.4)	172	7 (4.1)	201	1 (0.5)	175	4 (2.3)	<0.001
≥35	192	25 (13.0)	75	18 (24.0)	44	4 (9.1)	45	4 (8.9)	48	2 (4.2)	0.072
HIV and Other STDs coinfection	73	26 (35.6)	98	24 (24.5)	50	6 (12.0)	76	6 (7.9)	74	1 (1.4)	<0.001

*P values are from Cochran-Armitage trend analysis.

**Table 3:** HIV testing behavior and condom use among men who have sex with men in Chongqing.

Health behaviors	2006		2008		2010		2012		2013		*P* value*
	*N*	*n* (%)	*N*	*n* (%)	*N*	*n* (%)	*N*	*n* (%)	*N*	*n* (%)	
Ever received HIV testing in previous year	561	94 (16.8)	602	195 (32.4)	400	198 (49.5)	390	165 (42.3)	376	162 (43.1)	<0.001
Age (years)											
<25	158	29 (18.4)	322	96 (29.8)	184	84 (45.7)	144	58 (40.3)	153	63 (41.2)	<0.001
25–34	211	44 (20.9)	205	79 (38.5)	172	91 (52.9)	201	94 (46.8)	175	81 (46.3)	<0.001
≥35	192	21 (10.9)	75	20 (26.7)	44	23 (52.3)	45	13 (28.9)	48	18 (37.5)	<0.001
Condom use during last sexual intercourse with man	466	242 (51.9)	500	330 (66.0)	375	250 (66.7)	336	231 (68.8)	338	240 (71.0)	<0.001
Age (years)											
<25	135	80 (59.3)	268	175 (65.3)	171	117 (68.4)	124	84 (67.7)	137	98 (71.5)	<0.001
25–34	187	97 (51.9)	172	123 (71.5)	161	108 (67.1)	173	119 (68.8)	154	113 (73.4)	<0.001
≥35	144	65 (45.1)	60	32 (53.3)	43	25 (58.1)	39	28 (71.8 )	47	29 (61.7)	0.002
Consistent condom use in the last 6 months with men	466	115 (24.7)	500	185 (37.0)	375	155 (41.3)	336	150 (44.6)	338	162 (47.9)	<0.001
Age (years)											
<25	135	48 (35.6)	268	97 (36.2)	171	75 (43.9)	124	50 (40.3)	137	60 (43.8)	<0.001
25–34	187	45 (24.1)	172	71 (41.3)	161	64 (39.8)	173	82 (47.4)	154	88 (57.1)	<0.001
≥35	144	22 (15.3)	60	17 (28.3)	43	16 (37.2)	39	18 (46.2)	47	14 (29.8)	<0.001
Sex with women	561	185 (33.0)	602	95 (15.8)	400	45 (11.3)	390	45 (11.5)	376	33 (8.8)	<0.001
Condom use during last sexual intercourse with woman	185	57 (30.8)	95	43 (45.3)	45	15 (33.3)	45	22 (48.9)	33	17 (51.5)	0.008
Consistent condom use in the last 6 months with women	185	40 (21.6)	95	38 (40.0)	45	12 (26.7)	45	19 (42.2)	33	15 (45.5)	0.001
Commercial sex with men	561	39 (6.9)	602	51 (8.4)	384	16 (4.0)	376	14 (3.6)	367	9 (2.4)	<0.001
HIV/AIDS awareness rate	561	488 (87.0)	602	522 (86.7)	400	385 (96.3)	390	385 (98.7)	376	368 (98.0)	<0.001

*P values are from Cochran-Armitage trend analysis.
